# Bridging aging and colorectal cancer: synergistic roles of inflammaging and immunosenescence

**DOI:** 10.3389/fimmu.2026.1792954

**Published:** 2026-05-26

**Authors:** Silvere D. Zaongo, Qiyu Yang, Mei Han, Tianlan Xi, Zilang Luo, Xin Wang, Jiadan Yang, Jing Ouyang

**Affiliations:** 1 Department of Infectious Diseases, Chongqing Public Health Medical Center, Chongqing, China; 2 Clinical Research Center, Chongqing Public Health Medical Center, Chongqing, China; 3 Department of Drug Evaluation and Inspection, Southwest China Regional Center for Drug and Medical Device Evaluation and Inspection of National Medical Products Administration (NMPA), Chongqing, China; 4 Department of Pharmacy, The First Affiliated Hospital of Chongqing Medical University, Chongqing, China; 5 Pharmacy Department, Chonggang General Hospital, Chongqing, China

**Keywords:** aging, colorectal cancer, immunosenescence, inflammaging, mechanism

## Abstract

Colorectal cancer (CRC) is one of the most commonly occurring malignancies worldwide, with incidence and mortality rising sharply in older adults. While aging is increasingly recognized as a key risk factor for CRC, the fundamental immunological mechanisms which underlie this risk remain incompletely understood. Two interconnected processes, namely inflammaging and immunosenescence, appear central to this association. On the one hand, inflammaging, which is characterized by chronic low-grade inflammation in older individuals, fosters a tumor-promoting microenvironment through oxidative stress, genomic instability, and persistent cytokine activation. On the other hand, immunosenescence diminishes immune surveillance, reducing the clearance of premalignant cells and weakening responses to tumor progression and therapy. Together, these processes create an immunological framework that predisposes the aging colon to malignant transformation. This review synthesizes current knowledge of the cellular and mechanistic impacts of inflammaging and immunosenescence in CRC pathogenesis, highlighting their roles in shaping disease susceptibility in the elderly. These insights may guide future endeavor in biomarker discovery, prevention, and therapeutic intervention to mitigate the burden of CRC in aging populations.

## 1 Introduction

Colorectal cancer (CRC), also known as bowel cancer, colon cancer, or rectal cancer, is one of the most prevalent malignancies worldwide (1). According to the GLOBOCAN, an estimated 1.93 million new CRC cases were reported globally in 2020 (2). Geographic disparities exist, with higher incidence rates in developed regions (3). Pathologically, CRC usually originates from abnormal cell growths, known as polyps, within the colon or rectum. Although most polyps are benign, some may undergo malignant transformation over time. Notably, CRC predominantly affects adults, with incidence and mortality rising sharply in older individuals (4). Epidemiological data reveal that CRC accounts for a disproportionately high disease burden in the elderly, who exhibit a 1.5-fold higher incidence and a threefold higher mortality rate compared to younger populations (5). In the United States, right-sided CRC is more prevalent in elderly patients, with approximately half of those over 80 years old affected (6). Furthermore, older CRC patients have significantly lower 5-year survival rates compared to younger patients, particularly when comorbidities (e.g., cardiovascular disease, diabetes, among others) are present (7). Octogenarians undergoing CRC surgery face a 30% higher risk of postoperative mortality and reduced long-term survival, even with curative intent (8). Meanwhile, the incidence of early-onset CRC has been rising at an alarming rate. According to meta-analyses, several age-related factors, including hyperlipidemia, obesity, and alcohol consumption, have been identified as significant risk factors (9). Despite the clear association between aging and CRC, the immunological mechanisms which underlie this age-related susceptibility remain incompletely understood.

To address this critical gap, it is first necessary to clarify the concept of aging. Aging is defined as the gradual, time-dependent decline of physiological functions essential for survival and reproduction (10). It is a universal process shared across all living organisms, from individuals to entire species. From an immunological standpoint, aging presents two major challenges known as inflammaging and immunosenescence (11, 12), both of which have been strongly implicated in cancer development. Inflammaging is defined as a chronic, low-grade, systemic inflammatory state that progressively develops with advancing age (13). Unlike inflammation arising from infection, injury, or disease, inflammaging emerges intrinsically as a normal byproduct of the aging process. Immunosenescence, on the other hand, refers to the progressive, age-related deterioration of immune competence (14). Individuals experiencing immunosenescence display a reduced ability to mount effective immune responses, leaving them vulnerable to infections and malignancies (15). Interestingly, these preceding challenges observed during aging have profound implications in the pathogenesis of malignancies in general. Thus, inflammaging may foster a tumor-promoting environment within the colon by sustaining oxidative stress, genomic instability, and a proinflammatory cytokine milieu, thereby enhancing epithelial proliferation, invasion, and metastasis (16, 17). Meanwhile, immunosenescence may impair immune surveillance, diminishing the clearance of premalignant cells and weakening the capacity of the immune system to respond to both tumor development and therapeutic interventions (18). Together, it is likely that inflammaging and immunosenescence constitute an immunological cooperative framework through which aging predisposes to CRC initiation and progression. However, to fully validate this assertion, explicit evidence within the specific context of CRC is required. In addition, a deeper understanding of how age-related immunological processes contribute to CRC pathogenesis may not only shed light on disease mechanisms, but also expose new pathways for biomarker discovery, guide preventive strategies, and inspire therapeutic interventions aimed at mitigating the impact of aging on CRC incidence and mortality.

This review synthesizes current knowledge on how aging, through the intertwined processes of inflammaging and immunosenescence, contributes to CRC pathogenesis in the aging population. In particular, we explore the cellular and mechanistic impacts of inflammaging and immunosenescence that collectively foster an environment conducive to tumor emergence and promotion.

Herein, we aim to provide an integrated immunological framework specific to CRC, which systematically delineates the intertwined roles of inflammaging and immunosenescence in CRC pathogenesis among the aging population. This review specifically focuses on how these two age-associated processes jointly drive CRC initiation, progression, immune evasion, and adverse clinical outcomes in elderly individuals. In particular, we highlight the mechanistic interplay between chronic low-grade inflammation, impaired immune surveillance, tumor-promoting cytokine signaling, and age-related alterations within the colorectal tumor microenvironment.

## 2 Inflammaging and colorectal cancer

**Figure 1:**
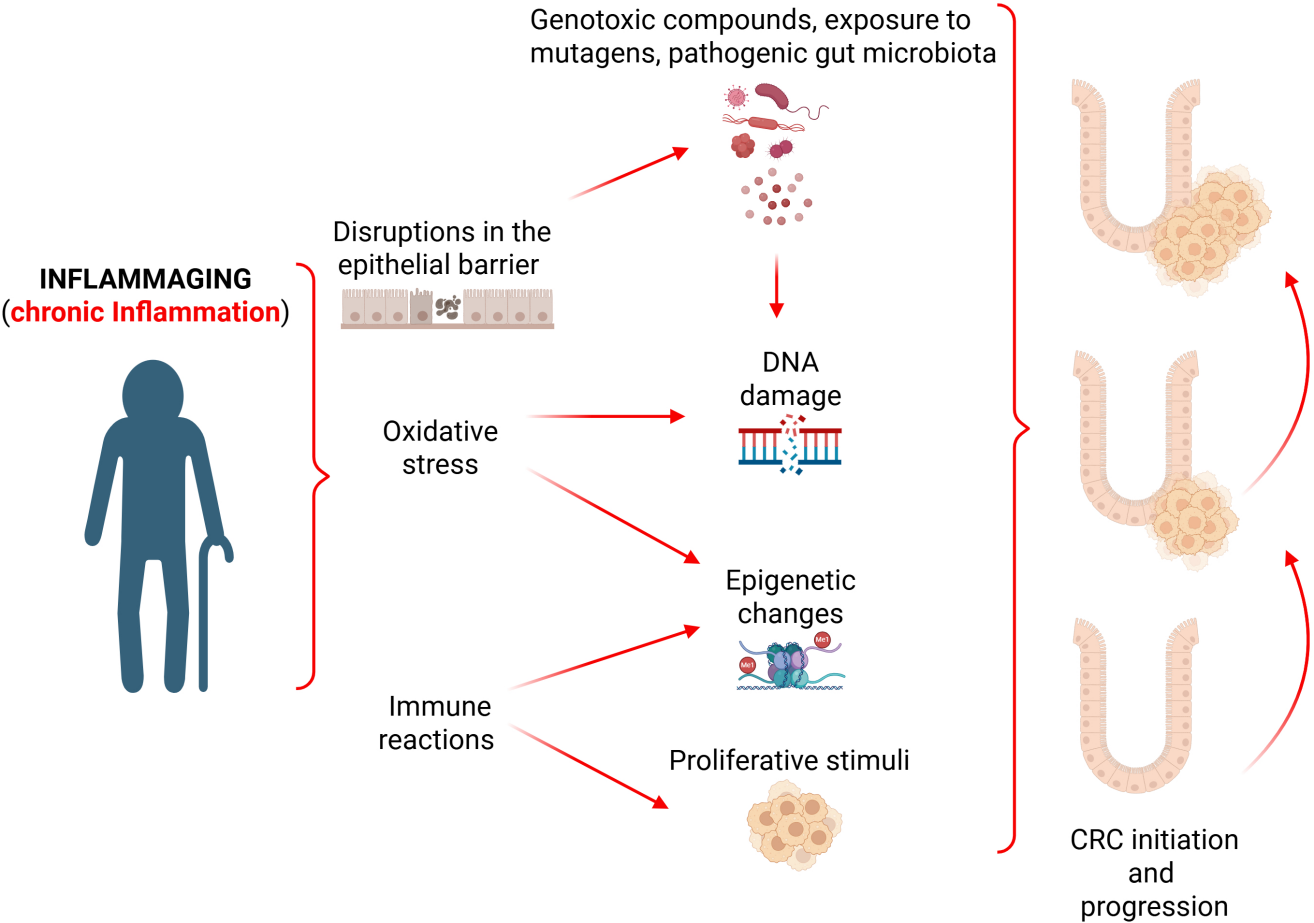
Chronic inflammation subsequent to inflammaging as a mechanistic link between aging and colorectal cancer.

Regarding the pathogenesis of CRC, the role of chronic inflammation is well established. Evidence indicates that poorly controlled inflammatory bowel disease, as well as chronic inflammation of the gastrointestinal tract driven by unhealthy dietary patterns (such as the Western-style diet) are the main risk factors for CRC (19, 20). Mechanistically, chronic inflammation contributes to tumor-initiation and tumor-promotion, which collectively represent two essential events required for the induction of tumor formation from normal cells (21). The roles of inflammation in the initiation and promotion of CRC in aging populations can therefore be elucidated through several key inflammation-related factors outlined below. A summary of these factors is provided in **Figure 1**.

### 2.1 Inflammation promotes the release of genotoxic compounds

Inflammation may drive the development of CRC through DNA damage (22). Indeed, inflammation is known to increase oxidative stress via recruited immune cells and/or via tissue-resident cells, which increases the release of genotoxic compounds (such as reactive oxygen and nitrogen species) into the tissue microenvironment (22). It is known that chronic inflammation may induce the expression of proinflammatory cytokines such as TNF-α, IL-6, and transforming growth factor-beta (TGF-β), which trigger the secretion of reactive oxygen and nitrogen species by non-phagocytic cells (23, 24). In the case of phagocytic cells, the release of reactive oxygen and nitrogen species is subsequent to their activation, which is mediated by inflammation (25, 26). Once reactive oxygen and nitrogen species are released in the colon, they may cause various types of DNA damage in the intestinal epithelial cells (IECs) (22). Specifically, DNA damage may include single-strand breaks, double-strand breaks, DNA or RNA lesions referred to as abasic sites [removal of a nitrogenous base (purine or pyrimidine)], and nucleotide modifications (21). Thus, during chronic inflammation, the release of reactive oxygen and nitrogen species by myeloid cells within the colon may directly induce the preceding forms of DNA damage, which in turn may trigger tumorigenesis in IECs (27). Notably, chronic inflammation may also promote epithelial-mesenchymal transition (EMT) by regulating the expression of TGF-β; this transition is a critical process driving tumor invasion and metastasis (28–30). Janney et al. (31) have reported that inflammation initiated by release of the preceding genotoxic compounds creates a mutagenic environment that fosters the initiation of CRC not only in IECs, but in intestinal stem cells as well. Specifically, the chronic intestinal inflammation created by induction of the release of genotoxic compounds may provoke an excessive tissue regenerative process by activation of tumor survival and growth pathways (32) (discussed further below), or by induction of the expression of anti-apoptotic proteins such as Bcl-2 and Bcl-xL (33). This is of significant importance as chronic intestinal inflammation may therefore (i) trigger the proliferation and clonal expansion of cells going through tumorigenesis, and (ii) provoke the de-differentiation of non-stem cells into stem-like cells to facilitate the regeneration of the damaged tissues (34–36). Hence, chronic inflammation may increase the mutational burden and the number of cells that are going through tumorigenesis. Indeed, on the one hand, compared to intestinal stem cells, non-stem cells are less resistant to high levels of replication stress and of DNA-damaging agents (37), while on the other hand, it has been demonstrated that the de-differentiation of post-mitotic (non-stem) cells may possess tumor-initiating capacity in mouse model (37).

### 2.2 Influence of inflammation on tumor signaling pathways

**Figure 2:**
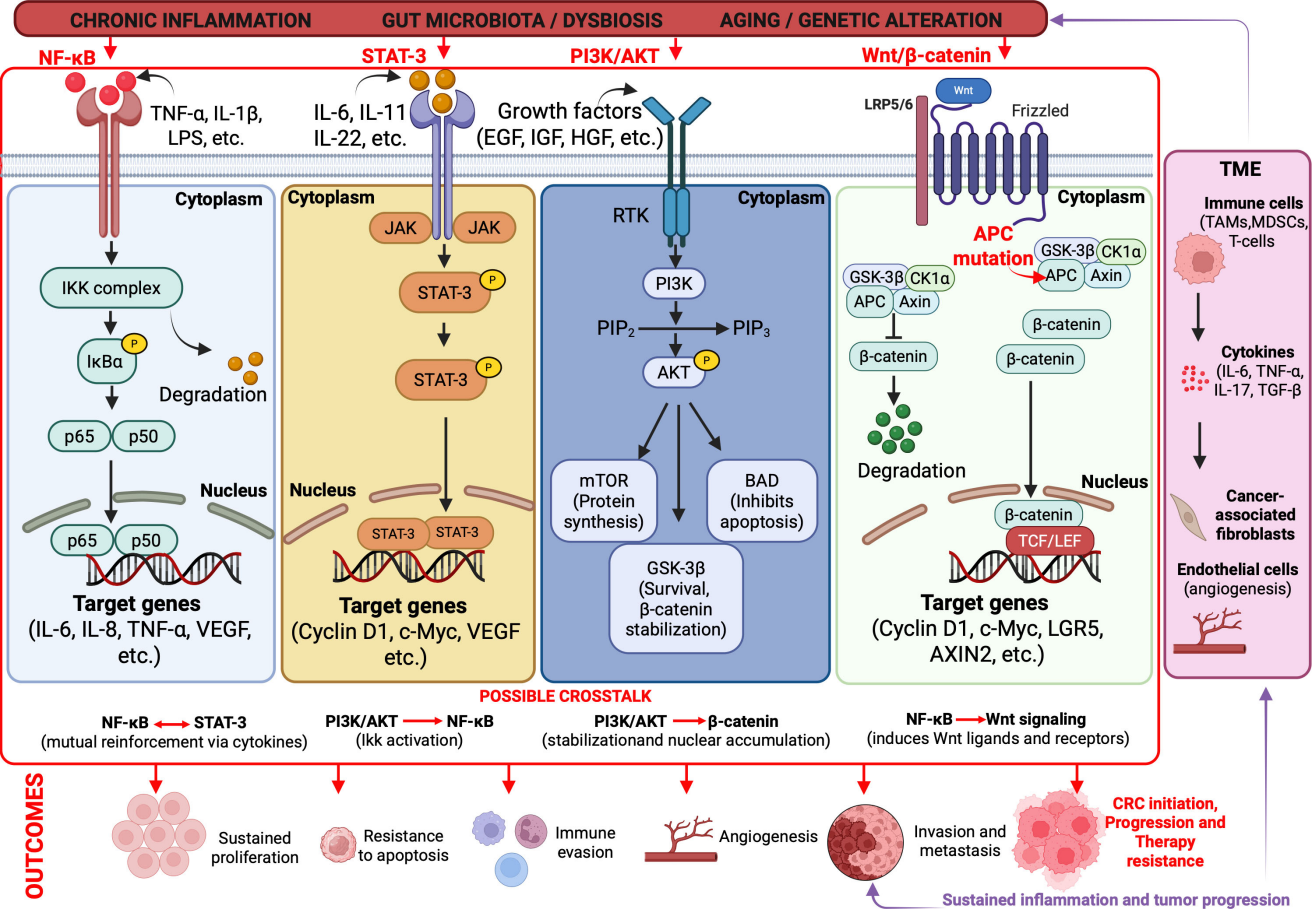
Key signaling pathways implicated in CRC arising from the convergence of chronic inflammation, microbial dysbiosis, and aging/genetic alterations. These factors collectively drive the activation of major oncogenic cascades, including NF-κB, STAT-3, PI3K/AKT, and WNT/β-catenin. Proinflammatory stimuli, such as TNF-α, IL-6, and microbial byproducts, trigger NF-κB and STAT-3 signaling, thereby upregulating the transcription of genes involved in cellular proliferation, survival, and immune evasion. Meanwhile, growth factor signaling activates the PI3K/AKT pathway, which enhances cell survival and promotes metabolic reprogramming. In parallel, mutations within the WNT pathway, most notably in the adenomatous polyposis coli (APC) gene, result in β-catenin accumulation and subsequent transactivation of genes that drive proliferation and maintain stemness. Extensive crosstalk among these pathways further amplifies tumor-promoting signals. Collectively, this interconnected signaling network contributes critically to colorectal tumor initiation, progression, and resistance to apoptosis.

Chronic inflammation affects key cytokine receptor-mediated signaling pathways known for their putative roles in tumor-initiation and tumor-promoting processes (38). As such, past studies have demonstrated that inflammation promotes CRC by inducing the activation of nuclear factor kappa B (NF-κB, a regulator of proinflammatory cytokines, cell adhesion molecules, and activity and development of immune cells) through downstream signaling of the tumor necrosis factor (TNF) receptor and IL-1 receptor (39–42). Similarly, Ibrahim et al. (43) have observed that the activation of NF-κB is positively associated with the regulation of phosphoinositide-3-kinase regulatory subunit 3 (PIK3R3), which in turn regulates phosphoinositide 3-kinase (PI3K), in patients with inflammatory bowel disease and CRC. Notably, PI3K activity is involved in multiple cell-signaling pathways responsible for cell survival, proliferation, and metabolism (44). PI3K may activate AKT, resulting in the overproduction of TNF-α, which exacerbates inflammation and may promote the transition from ulcerative colitis (UC) to CRC (45). Furthermore, the PI3K/AKT pathway may (i) inactivate the production of proapoptotic proteins [for example, Bcl-2 associated agonist of cell death (Bad) and Caspase-9 (46)], (ii) stimulate angiogenesis by promoting the production of vascular endothelial growth factor, therefore ensuring adequate blood supply to the tumor (46), and (iii) suppress the functions of Tregs (promoting evasion from immune surveillance) or influence the polarization of macrophages (which support tumor growth and metastasis) (47, 48). In addition to the NF-κB and PI3K/AKT pathways, inflammation may also trigger gp130-dependent activation of the transcription factor STAT-3 via downstream signaling induced by IL-6 and/or IL-11 (49–51). Evidence from genetic mouse models indicates that in the context of gastrointestinal tumor progression, IL-11 produced by cancer-associated fibroblasts and myeloid cells activates the STAT-3 signaling pathway more strongly than IL-6 (51). Interestingly, in a cohort of 375 colon cancer patients, Heichler et al. (52) found that IL-11-driven STAT activation in cancer-associated fibroblasts is strongly associated with a poor prognosis. Moreover, it is important to note that gain-of-function mutations in TP53 arise at very early stages of CRC and are known to potentiate TNF, NF-κB, and STAT-3 signaling (53, 54). Furthermore, in the context of cancer, it is well known that AKT and extracellular signal-regulated kinase 1/2 (ERK1/2) activation promotes cell proliferation either by (i) modifying the function of proteins containing oxidation-sensitive cysteine residues or (ii) enhancing the expression of proliferation-associated genes, including c-Myc and cyclin D1, thereby driving cell cycle progression and tumor growth (55, 56). The activation of ERK1/2 is involved in cell survival through the expression of Bcl-2 and Bcl-xL (33). Inflammation may also influence the activation of AKT and ERK1/2 in CRC. Lastly, the Wingless and Int-1 (WNT) pathway, which regulates the inflammatory cascade response and oxidative stress, has been associated with the development of CRC through the activity of β-catenin (57). In brief, β-catenin mediates WNT signal transduction to activate the expression of genes resulting in (i) uncontrolled cell division and tumor growth, (ii) maintenance of the self-renewal capacity of cancer stem cells and facilitation of the epithelial-mesenchymal transition, and (iii) the support of tumor growth, as well as protection of the tumor from immune attacks (58–60). The key mechanisms involved in CRC are summarized in **Figure 2**.

### 2.3 Influence of inflammation on the expression of genes associated with CRC

It is known that genes such as NOTCH and p53 are involved in the development of CRC. Indeed, NOTCH signaling is often activated to promote tumor growth and metastasis, while the tumor-suppressor gene p53 is frequently mutated or lost, leading to unchecked growth and invasiveness (61). Intriguingly, inflammation may induce epigenetic mechanisms that result in silencing of critical tumor suppressing genes (62). Indeed, by inducing the release of proinflammatory elements such as IL-1β, IL-6, and TNF-α, chronic inflammation may control the expression of DNA methyltransferases (DNMT)-1 and 3. Thus, inflammation may induce changes in methylation and the expression pattern of NOTCH and p53, which may influence the development of CRC (63, 64). For instance, the methylation in the promoter region of the p53 gene may induce its downregulation, leaving the initiation and proliferation of CRC cells uncontrolled. It has also been demonstrated that NOTCH signaling subsequent to inflammation leads to highly penetrant metastasis in KRAS-driven CRC through TGF-β-dependent neutrophil recruitment (65). Similarly, in the context of inflammation, Varga et al. (66) have observed that NOTCH signaling may drive invasion and metastasis in a consensus molecular subtype 4 (CMS4) tumor model. Additionally, the development of CRC, lung, and pancreatic cancers in mice has been linked to overexpression of IL-1α, a proinflammatory cytokine associated with aging, following a decline in DNMT3A expression (67). Anti-IL-1α administration suppressed tumor cell growth by blocking the IL-1α/IL-1R1 axis. More importantly, profiling of IL-1α mRNA levels in tumors from patients aged over 70 years, compared to younger patients, identified IL-1α as a potential predictor of lung cancer risk (67). Furthermore, investigations by Sun et al. (68) demonstrated that IL-1α mRNA expression in human CRC tissues is (i) significantly elevated, (ii) positively correlated with clinical stage and metastatic status, and (iii) may therefore predict CRC initiation and progression.

### 2.4 Inflammation increases prostanoids

During chronic inflammation, prostanoids (lipid mediators) are produced in a cyclooxygenase 1 (COX1)-dependent and COX2-dependent manner (69, 70). Notably, evidence indicates that prostanoids have a major impact on CRC immunopathogenesis (71, 72). As such, to promote the initiation and growth of CRC, Wang and Dubois (72) have reported that prostaglandin E_2_ (PGE_2_, the most abundant prostanoid in CRC) (i) induces the expression of DNMT1 and DNMT3B, and (ii) favors the clonal expansion of cancer stem cells. Specifically, the clonal expansion of cancer cells is mediated via the activation of NF-κB signaling (72). Similarly, studies have observed that PGE_2_ may enable immune evasion by affecting macrophage polarization (71), inducing the differentiation of myeloid-derived suppressor cells (MDSCs) and Treg cells (73), and inhibiting the natural killer (NK)-cell-dependent remodeling of the tumor microenvironment (TME) (74).

### 2.5 Influence of inflammation on epigenetic silencing of cancer-related pathways

Chronic inflammation may be associated with CRC through its capacity to influence the expression and the activity of microRNAs and long non-coding RNAs (lncRNAs) targeting two critical drivers of CRC, namely the WNT pathway and the Hippo pathway (75). These epigenetic elements may also regulate the signaling pathways of p53, STAT-3, and NF-κB, as observed by several research teams (76–80). For instance, in patients with ulcerative colitis, Geng et al. (81) have observed that the expression of lncRNA H19 is significantly elevated. They observed a similar pattern of lncRNA H19 expression in murine models of lipopolysaccharide-induced sepsis and dextran sulfate sodium (DSS)-induced colitis (81). Mechanistically, the increased levels of lncRNA H19 in IECs are attributable to the production of IL-22 by neutrophils, innate lymphoid cells, Th17, and T_h_22 cells (82–84). As shown by Geng et al. (81), lncRNA H19 acts by (i) enhancing the proliferation of IECs and (ii) increasing the regeneration of the colonic epithelium. Therefore, as observed in the context of DSS-induced colitis, it is likely that inflammation-mediated increases in lncRNA H19 levels may favor the proliferative capacity of malignant cells in the context of CRC as well.

### 2.6 Inflammation-induced gut dysbiosis as a driver of CRC

Inflammaging disrupts the inherent intestinal microbiome balance and may therefore provoke gut dysbiosis. As demonstrated via examination of stool sampled from patients with CRC versus healthy patients, the gut dysbiosis in individuals with CRC is characterized by a reduction of beneficial microbes, while pathogenic and proinflammatory organisms are promoted (85–87). Several studies have provided interesting evidence regarding the specific mechanisms which potentially associate gut dysbiosis to CRC development. Firstly, a genotoxicity exerted by bacteria and their metabolites may transform IECs and establish tumorigenesis. For instance, it has been demonstrated that *Escherichia coli* (*E. coli*) harboring the genomic island polyketide synthase (pks+ E. coli) via the production of colibactin may potentially influence the production of mutagenic DNA (88). Intriguingly, pks+ *E. coli* has been found to be abundant in preclinical models of CRC and human tumor tissues (89), suggesting its strong potential association in the development of CRC. Besides, Kadosh et al. (90) demonstrated that gallic acid produced by the gut microbiome may trigger the transition from a tumor-suppressive to an oncogenic environment in a mouse model. Secondly, as summarized by Burgos-Molina et al. (46), the destruction of the superficial gut barrier due to gut dysbiosis may further amplify inflammation, thereby fostering tumorigenesis. In other words, the gut permeability and exposure to microbes and microbial products (e.g., LPS) may favor the development of CRC by prompting the recruitment of myeloid cells into the TME (46). Similarly, commensal bacteria may penetrate tumor tissue directly, activating infiltrating myeloid cells to produce inflammatory cytokines, thereby amplifying CRC development (46). Thirdly, CRC may be triggered by pathogenic bacteria such as *Fusobacterium nucleatum* (*F. nucleatum*) due to their capacity to induce inflammation. Indeed, evidence indicates that patients with CRC have a significant augmentation of *F. nucleatum* (91, 92), which is associated with a poorer prognosis (93). *F. nucleatum* amplifies the expression of oncogenes (including cyclin D1 and MYC) and proinflammatory signals (TNF and IL-17) by triggering β-catenin signaling (94). Interestingly, *F. nucleatum* is capable of modulating the onset of CRC via its interactions with both tumor cells and immune cells present within the TME. As such, it has been demonstrated that *F. nucleatum* triggers tumor progression by (i) favoring the recruitment of MDSCs, tumor-associated neutrophils (TANs), TAMs, and immature dendritic cells (DCs) possessing immunosuppressive capabilities (95) and (ii) triggering the expression of specific chemokines (CXCL8 and CXCL1) that amplify the migratory capability of tumor cells and reinforcing their metastatic potential (96). Besides, by engaging with T-cell immunoreceptor with Ig and ITIM domains (TIGIT, an immune checkpoint receptor), *F. nucleatum* may (i) attenuate T-cell activation and (ii) impair natural killer (NK) cell-mediated clearance of colon tumor cells (97). In addition to *F. nucleatum*, some studies have highlighted the role of enterotoxigenic *Bacteroides fragilis* in the development of CRC. Evidence indicates that enterotoxigenic *Bacteroides fragilis* influences the recruitment of other bacterial species to form biofilms that coat colorectal adenomas and tumors (98). Moreover, enterotoxigenic *Bacteroides fragilis* induces an inflammatory response within colonic epithelial cells, which may facilitate the progression of CRC (99). As an example, enterotoxigenic *Bacteroides fragilis* may recruit MDSCs to the TME in order to suppress anti-tumor immune responses (100).

Notably, pathogenic fungi in contaminated food, including *Aspergillus* spp., *Penicillium* spp., and *Fusarium* spp. may directly promote carcinogenesis through the production of mycotoxins (i.e., aflatoxins, patulin, trichothecenes, and fumonisin) in the intestinal tract (101). Among the mycotoxins is sterigmatocystin, which in the presence of *Helicobacter pylori*, may promote gastric carcinomas and intestinal metaplasia (102, 103). To a larger extent, it has been suggested that the abundance of a few species belonging to the *Aspergillus, Candida, Saccharomyces, Trichosporon, Cladosporium, Malassezia, Cryptococcus, and Galactomyces* genera may be associated with the development of CRC (104). Notably, as suggested by Schmitt and Greten (21), the influence of commensal fungi on the development of CRC requires further clarification. As is known, the recognition of fungal cell wall components by pattern recognition receptors (PRRs) on antigen-presenting cells may activate tyrosine kinases and trigger inflammatory responses. Thus, the production of proinflammatory cytokines may occur through the activation of the NF-kB pathway. Hence, through their potential capacity to promote inflammation and activation of the NF-kB pathway (which, among other pathways, is itself involved in the pathogenesis of cancers), it is reasonable to postulate that commensal fungi may also play a critical role in the onset and evolution of CRC, particularly in older individuals.

**Figure 3:**
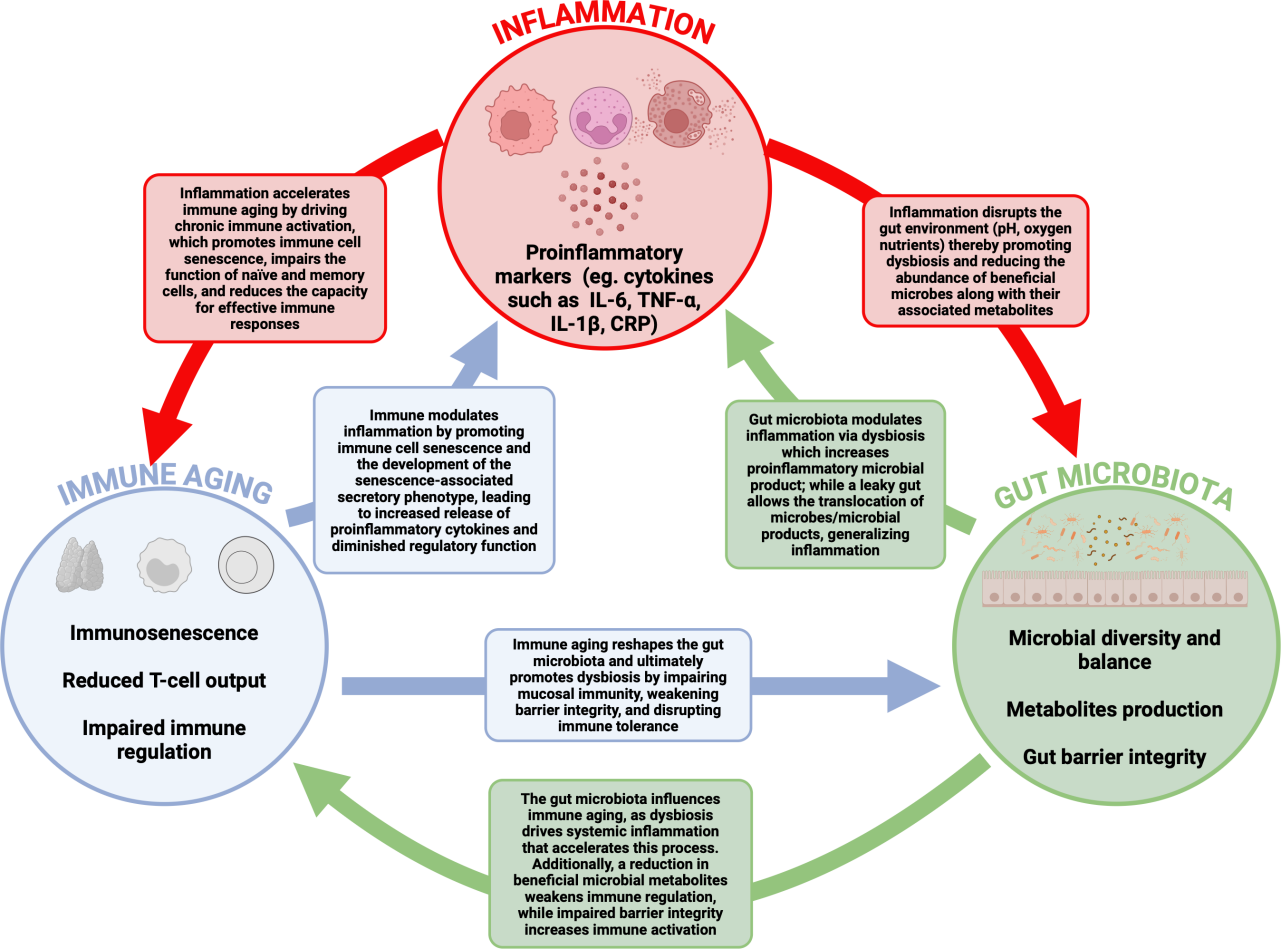
Interactions among inflammation, immune aging, and the gut microbiome. These interrelationships can be viewed as dynamic, bidirectional processes that form a self-perpetuating cycle, influencing health and disease, such as CRC, across the lifespan. Red arrows indicate the dynamic influences of inflammation, blue arrows represent the dynamic influences of immune aging, and green arrows denote the dynamic influences of the gut microbiome.

A diagram illustrating the interplay among inflammation, immune aging, and the gut microbiome is presented in **Figure 3**. A deeper understanding of these interactions may further elucidate how aging contributes to the development of CRC.

## 3 Immunosenescence and colorectal cancer

Aging is the primary driver of immunosenescence (105), which is characterized by significant alterations of immune cells, impaired immune responses, and chronic inflammation (18, 106). Thus, immunosenescence, by itself, may fuel the inflammaging process (18) and thus potentially promote the development of CRC via the mechanisms described above in Chapter 2. More specifically, immunosenescence may be driven by age-related factors such as (i) antigenic stimulation resulting from infections (107) or the TME (108), (ii) destruction of the telomere-telomerase system (109, 110), (iii) involution of the thymus (111–115), and (iv) chronic inflammation (116). Beyond these factors, sex may also influence immunosenescence (117). Indeed, as suggested by Calabro et al. (117), sex may influence immunosenescence via hormonal, genetic, and sociocultural factors (differences in lifestyle and environmental exposure) that shape immune responses differently in men and women.

Pereira et al. (118) have demonstrated that senescent cells escape immune elimination by releasing senescence-associated secretory phenotype (SASP) factors such as IL-6. These factors increase HLA-E expression, thereby inhibiting the ability of NK cells and T-cells to clear premalignant lesions. Cellular senescence is (i) marked by abnormal alterations in cell shape, gene activity, chromatin structure, and metabolic processes and (ii) triggered by persistent stimulation from the surrounding microenvironment (119, 120). Immunosenescence broadly affects immune cell. Therefore, understanding how immunosenescence alters innate and adaptive immune cells, and how this may trigger or favor the development of CRC, requires a careful review of the contemporary literature. **Table 1** summarizes the major links between immunosenescent cells and CRC.

**Table 1:** Major links between immunosenescent cells and colorectal cancer (CRC).

Senescent immune cells	Changes	Effects associated with the initiation and progression of CRC	References
Macrophages	Antigen-presenting ability ↓; DPP7 expression and fatty acid β-oxidation ↑; secretion of proinflammatory cytokines (e.g., IL-6) and angiogenic factors ↑.	1. Induces CD8+ T-cell exhaustion within the tumor microenvironment. 2. Promotes angiogenesis and nutrient supply to support tumor growth. 3. Remodels the extracellular matrix to facilitate CRC invasion and metastasis. 4. Drives chronic inflammation that underpins CRC development.	(121–123)
Dendritic cells	Antigen uptake and processing capacity ↓; impaired maturation, with MHC II and co-stimulatory molecules (e.g., CD80) ↓; dysregulated cytokine secretion; ability to prime naive T-cells ↓.	1. Breaks adaptive immunity initiation, failing to mount effective anti-CRC T-cell responses. 2. Promotes immune tolerance by inducing T-cell anergy. 3. Exacerbates susceptibility by contributing to inflammaging via imbalanced cytokines. 4. Limits efficacy of DC-based cancer vaccines.	(15, 124, 125)
Natural killer cells	Total number and CD56bright regulatory subset ↓; CD56dim CD57+ terminally differentiated subset ↑; Activating receptors (e.g., NKG2D) ↓; Inhibitory receptors (e.g., KIR) ↑; Cytotoxicity and IFN-γ secretion ↓; telomere shortening.	1. Compromises innate immune surveillance, allowing CRC cells to evade early elimination. 2. Accelerates CRC progression due to impaired recognition of malignant cells. 3. Reduces potential efficacy of immunotherapies leveraging NK cell activity.	(126, 127)
Neutrophils	Skewing toward a pro-tumor N2 phenotype (characterized by CD16 ↑); NETosis (Neutrophil Extracellular Trap formation) ↑; Arginase-1 activity ↑; Altered cytokine secretion profile.	1. Promotes tumor progression via immunosuppression (e.g., by arginine depletion). 2. Facilitates metastasis through ECM remodeling and providing a pro-metastatic niche. 3. Sustains inflammation and can directly damage surrounding tissues, potentiating carcinogenesis.	(18, 128)
Myeloid-derived suppressor cells	Frequency and suppressive activity ↑ with aging; Expansion of polymorphonuclear (PMN-MDSC) and monocytic (M-MDSC) subsets; Expression of Arginase-1, iNOS, and ROS ↑; Secretion of IL-10 and TGF-β ↑.	1. Potently inhibits CD8+ T-cell activity and promotes regulatory T-cell expansion. 2. Creates a dominant immunosuppressive niche in the tumor microenvironment, fostering CRC immune evasion. 3. Correlates with advanced disease stage and poorer response to immunotherapy.	(18, 129)
T-cells	Thymic involution leading to naive T-cells ↓; CD28- senescent T-cells and memory T-cells ↑; TCR diversity and proliferative capacity ↓; inhibitory receptors (e.g., Tim-3) ↑ and activation molecules ↓; effector cytokine (e.g., IFN-γ) secretion ↓.	1. Weakens immune surveillance, impairing effective recognition and clearance of premalignant and CRC cells. 2. Promotes tumor immune escape due to reduced cytotoxicity. 3. Diminishes responsiveness to tumor progression and immunotherapy. 4. Synergizes with inflammaging to foster a pro-tumorigenic microenvironment in the aging colon.	(130–132)
B cells	Expansion of age-associated B cells (ABCs, CD27-CD38-); autoantibody production ↑; secretion of proinflammatory cytokines (e.g., IL-6, TNF-α) ↑.	1. Sustains chronic low-grade inflammation (inflammaging), providing a pro-tumorigenic milieu. 2. May support tumor-associated fibroblast activation and tissue remodeling. 3. Contributes to immune dysregulation rather than effective anti-tumor humoral response.	(133, 134)

↓: reduce, reduces, or reduced; ↑: increase, increases, or increased.

### 3.1 The role of senescent innate immune cells in CRC

#### 3.1.1 Macrophages

An intriguing phenomenon, referred to as a shift to a pro-tumor phenotype, may explain how macrophage senescence may favor the development of CRC. In normal tissues, macrophages are usually polarized toward a proinflammatory state. However, within the TME, macrophages more often exhibit the functionality of promoting tumor growth through extracellular matrix remodeling, angiogenesis, and suppression of immune responses. In elderly individuals, the preceding trend favoring macrophages with tumor-promoting properties has also been observed (18). Besides, macrophages in elderly individuals show a decline in their antigen-presenting capacity due to the reduced expression of major histocompatibility complex (MHC) class II and co-receptors (135, 136). This is a profile well-known for its positive contribution to cancer development. Some other macrophages are also increasingly recognized as having a dual role. Although these macrophages act against tumors, their prolonged cytotoxic activity may drive the emergence of more aggressive and immune-resistant tumor clones, ultimately aiding tumor progression (137, 138). With age, macrophages progressively acquire characteristics marked by (i) permanent cell-cycle arrest [e.g., upregulation of p16 inhibitor of cyclin-dependent kinase 4a (p16^INK4a^)] (139, 140), (ii) reduced functions such as phagocytosis (141), and (iii) distinct features associated with SASP (142). A review by Wang et al. (143) provides a broader view on the genes associated with SASP in senescent macrophages. Briefly, by promoting the secretion of factors including IL-6, IL-8, IL-1α, IL-10, chemokine (C-C motif) ligand 18 (CCL18), matrix metalloproteinases (MMPs), TGF-β1, and vascular endothelial growth factor (VEGF), among others, senescent macrophages may promote cancer development (144). Similarly, senescent macrophages characterized by CXCR1^high^ (tissue-resident alveolar macrophages) have been observed to accumulate in lung adenocarcinoma lesions and to promote the formation of lung adenomas. A further look at this type of macrophage in the context of CRC is needed. In mouse lung adenocarcinoma, CXCR1^high^ macrophages have been shown to inhibit the ability of cytotoxic T-cells to kill cancer cells (145). In humans, the possibility that they also promote CRC via similar inhibitory effects warrants investigation.

Using single-cell RNA sequencing of 51 CRC samples, Ding et al. identified senescence-associated macrophages (SAMs) as key players in tumor-stroma crosstalk (146). Mechanistically, urokinase plasminogen activator and its receptor (PLAU/PLAUR) activate NF-κB through NFKBIA phosphorylation, thereby driving CRC proliferation, metastasis, and heterogeneity. To silence PLAU expression, Ding et al. (146) engineered a photo-crosslinkable siPLAU gene-patch using nanomaterial carriers. Both *in vitro* and *in vivo*, this patch suppresses NF-κB/epithelial-mesenchymal transition (EMT) signaling, reduces tumor growth and metastasis, and promotes apoptosis. Additionally, the patch enhances the infiltration of CD4+/CD8+ T-cells, IFN-γ+ cytotoxic T-cells, and proinflammatory macrophages (146). In another study, Yu et al. (147) highlighted the importance of macrophage senescence in the development of CRC. Indeed, this study developed a seven-gene [viz., prostaglandin E2 receptor 2 (PTGER2), fibroblast growth factor 2 (FGF2), insulin-like growth factor-binding protein 3 (IGFBP3), angiopoietin-Like 4 (ANGPTL4), dickkopf-related protein 1 (DKK1), WNT16, and secreted phosphoprotein 1 (SPP1)] senescence-related prognostic model for CRC using TCGA and GEO datasets. The model effectively stratifies patients into high- and low-risk groups, with low-risk patients showing enhanced survival and higher predicted immunotherapy response. Functional analyses linked differentially expressed genes to extracellular matrix interactions and focal adhesion pathways. High-risk tumors displayed macrophage enrichment and an immunosuppressive microenvironment, with SPP1+ macrophages strongly associated with senescent tumor cells. Further analysis revealed that SPP1+ macrophages highly express senescent factors such as CCL20, CXCL1, MMP12, CXCL10, IL6, and CCL5. These observations suggest that senescence may favor the selection of SPP1+ macrophages, which play key roles in CRC prognosis and tumor progression (147). Interestingly, it has been demonstrated that SPP1+ TAMs have a proangiogenic signature and are more likely to engage in crosstalk with cancer-associated fibroblasts (CAFs) and endothelial cells (148, 149). Furthermore, a strong infiltration of SPP1+ macrophages is associated with poor disease prognosis and resistance to immunotherapy (148, 149). In addition, experimental studies using CT26 mouse CRC cells showed that tumor-derived IL-6 may induce macrophage senescence, suggesting a feedback loop in which cancer cells drive nearby macrophages into a dysfunctional, pro-tumor state (150). Inflammation-driven expression of IL-6 in aging individuals may therefore favor the onset of senescent macrophages, which in turn may promote CRC.

#### 3.1.2 Dendritic cells

Senescent dendritic cells (DCs) may play an important role in the development of CRC through four major pathways. Firstly, senescent DCs are dysfunctional. Several studies have demonstrated that DCs in the CRC TME are impaired in their ability to migrate and mature effectively. This impairment correlates with poor T-cell priming and worse clinical prognosis (151–153). Si et al. (154) showed that the programmed death-ligand 1 (PD-L1) and STAT-3 signaling pathways are critical for senescence induction in DCs mediated by tumor-derived γδ Treg cells, leading to loss of their functions. Although these dysfunctional DCs are often attributed to tumor-derived immunosuppressive signals, age-related or stress-induced senescence may be an underappreciated contributing mechanism (155). Secondly, senescence impairs antigen-presenting cell function. In tumor and aging models, senescent antigen-presenting cells (including DCs) lose effective antigen processing and T-cell-stimulatory capacity, while acquiring a SASP (156). This includes the secretion of inflammatory cytokines driving immunosuppressive functions (e.g., IL-6, IL-10), which may feed chronic inflammation and blunt cytotoxic T-cell responses. Observations by You et al. (157) indicate that DCs from aged subjects exhibit reduced expression of co-stimulatory molecules such as CD80 and CD86, as well as decreased production of proinflammatory cytokines such as IL-12. Additionally, senescent DCs display impaired phagocytic activity, reduced capacity to produce IFN, and diminished chemotaxis (158). Notably, the latter two features significantly compromise the ability of DCs to elicit protective T-cell responses (158, 159). Thirdly, the senescent DC is a key player in senescence-driven immunotherapy resistance. Indeed, Si et al. (154), observed that DCs senescence occurs through the PD-L1 and STAT-3 signaling pathways. Based on this observation, they demonstrated that blocking these pathways promotes HER2 tumor-specific immune responses and immunotherapy efficacy in a humanized mouse model. Recent evidence also suggests that accumulation of senescent myeloid cells (including DCs and macrophages) within the TME may compromise immunotherapy by limiting the infiltration and activation of CD8+ T-cells (160–162). If senescent DCs are present in CRC tumors, they may directly contribute to this resistance by failing to prime T-cells or by creating a cytokine milieu unfavorable to effective anti-tumor immunity. Fourth and lastly, senescent DCs themselves may be part of the aged TME. In CRC patients, the TME is increasingly recognized as a disease-promoting and treatment-resistant environment (163). Thus, one may reasonably hypothesize that senescent DCs within older individuals (or in response to genotoxic stress, chemotherapy, or chronic inflammation) contribute to this aged immune phenotype, further weakening immune surveillance and facilitating tumor progression. For instance, inflammasome activation (e.g., NLRP3) in senescent DCs amplifies IL-1β release, driving chronic inflammation and CRC progression in aging mice, while NLRP3 inhibition attenuates tumor growth (164). Altogether, the preceding features of senescent DCs may lead to weaker priming of anti-tumor T-cells and increased tolerance toward transformed epithelial cells in the colon, therefore favoring CRC development.

#### 3.1.3 Natural killer cells

To promote CRC development, senescent NK cells may contribute through (i) reduced absolute counts or (ii) loss of cytotoxic and immunoregulatory functions, accompanied by immune-evading phenotypes. On the one hand, aged mice exhibit lower NK cell counts across all lymphoid organs (165). Similarly, CRC patients show significantly reduced NK cell frequency and absolute counts, specifically the anti-tumor CD56^dim^CD16^+^ NK cell subset, compared to healthy controls (166). In contrast, the CD56^bright^ NK and CD56^dim^CD16^-^ NK cell subsets, which lack effective anti-tumor activity, are more frequent in CRC patients than in healthy individuals (166). On the other hand, tumor-infiltrating NK cells from CRC patients frequently show diminished expression of activating receptors (including NKG2D and natural cytotoxicity receptors such as NKp46 or NKp30) (167–171) and reduced levels of perforin and granzyme B (166, 172). Additionally, NK cells from older individuals secrete lower amounts of IL-2 and IFN (18). IL-2 is essential for the proliferation of T-cells and NK cells. Thus, its reduced expression impairs immune responses (173). Notably, combining cetuximab with IL-2 or IL-15 may activate dysfunctional NK cells in CRC patients, suggesting a therapeutic role for IL-2 in senescent NK cells (168). Furthermore, diminished IFN levels promote tumor development in older adults, as IFN usually exerts anti-tumor and immunomodulatory effects (18).

Senescent NK cells are associated with deeper tumor invasion, lymph node metastasis, and poorer prognosis (174, 175). To illustrate this, single-cell and tumor-landscape studies into CRC cases reveal subsets of tumor-associated NK cells with dysfunctional or “exhaustion/senescence-like” transcriptional signatures (e.g., PD1+, RGS1+), which correlate with impaired T-cell recruitment and resistance to immunotherapies (170, 176). These observations indicate that accumulation of senescent NK cells undermines both innate and adaptive anti-tumor responses (170, 176). Such NK cells exhibit a senescence-associated loss of killing capacity, limiting their ability to eliminate CRC cells (168, 172). In parallel, senescent NK cells may secrete proinflammatory (IL-6) and immunosuppressive (IL-10) cytokines as part of their SASP. Collectively, senescent NK cells may sustain chronic inflammation while suppressing effective anti-tumor responses, thereby promoting a tumor-permissive microenvironment in the colon (177). The TME itself may accelerate NK cell senescence through hypoxia and persistent TGF-β exposure. Indeed, the hypoxic (TGF-β-rich) milieu of many CRC tumors suppresses NK cytotoxic machinery and surface-activating receptors, potentially driving senescence-like programs in innate lymphocytes and promoting immune evasion (178, 179). Lastly, tumor-intrinsic factors may also contribute. For example, ZNF217 is upregulated in CRC-associated NK cells, where it suppresses NK tumoricidal activity. Meanwhile, metabolic stress within the TME restricts NK effector function and favors senescence-like phenotypes (180, 181).

#### 3.1.4 Neutrophils

Senescent neutrophils have emerged as key drivers of the TME remodeling and cancer progression. This may be attributed to their reduced phagocytic activity, impaired chemotaxis, and dysfunctional toll-like receptors (TLRs) (128, 182). The proportion of senescent neutrophils correlates positively with cancer progression, and their selective removal significantly reduces tumor growth in mouse models of prostate cancer (183). Moreover, senescent neutrophils inhibit CD8+ T-cell aggregation and activity, thereby diminishing T-cell-mediated tumor elimination (184). Through SASP factors such as IL-6, senescent neutrophils may activate the JAK/STAT-3 signaling pathway within the TME (185–187), which drives tumor cell proliferation, survival, invasiveness, and metastasis (188). These properties likely contribute to colorectal carcinogenesis. One study further reports that tumor‐associated neutrophils acquire prolonged survival and a SASP, allowing them to persist in the TME and sustain chronic inflammation and immunosuppression (189). Specifically, senescent neutrophils secrete elevated levels of proinflammatory cytokines, chemokines, and miRNAs (190). These factors impair phagocytosis and ROS production (27, 191) while recruiting additional immunosuppressive cells such as MDSCs and Tregs (189). Consequently, these neutrophils may stimulate angiogenesis and extracellular matrix remodeling, thereby facilitating tumor growth and metastasis (192–194). A distinct subset of senescent neutrophils, characterized by a CXCR4^high^CD62L^low^ phenotype, accumulates in the TME and plays a major role in metastasis development. This phenotype results from active SIRT1 expression in neutrophils, originally induced by nicotinamide phosphoribosyltransferase (NAMPT) secreted by tumor cells. SIRT1 expression promotes the release of mitochondrial DNA, leading to the formation of mitochondrion-dependent nonlethal neutrophil extracellular traps (NETs), which capture tumor cells and enhance their migration (195). In another study, chemotherapy-induced senescent neutrophils released exosomes rich in regulatory small RNAs, conferring chemoresistance and promoting EMT in cancer cells (196). Collectively, in the pathogenesis of CRC, senescent neutrophils may not be passive bystanders but may act as potent drivers of inflammation, immune evasion, metastasis, tissue remodeling, and therapeutic resistance.

#### 3.1.5 Myeloid-derived suppressor cells

MDSCs, defined as cells of neutrophil and monocyte lineages, exert potent immunosuppressive activity. Although studies on senescent MDSCs in CRC are still limited, broader senescence literature offers relevant insights. As such, senescent MDSCs play a critical role in tumor progression and immune evasion through their SASP (including IL-6, IFN-γ, IL-1β, TNF-α, VEGF, and GM-CSF) and altered functional state (197, 198). These cells may enhance cancer cell survival and proliferation by activating the androgen receptor pathway (199) or diminish immunotherapy effectiveness via the NF-kB pathway (200). Therapy-induced senescence (TIS) may exacerbate this problem. Indeed, senescent cells lingering after genotoxic treatments release SASP factors that recruit and activate suppressive myeloid populations, including MDSCs, thereby blunting anti-tumor immunity and potentially contributing to therapeutic resistance (201). Besides, in tumor-bearing and aged mice, senescent MDSCs expand in response to SASP stimuli (e.g., IL-6 and GM-CSF, among others), correlating with immune suppression and poorer outcomes (13, 202, 203). In parallel, the expansion of senescent MDSCs may suppress T-cell activity and release tumor-promoting factors. For instance, MDSCs accumulate within the TME of elderly CRC patients. Once there, they suppress effector T-cells and foster an immunosuppressive milieu through the secretion of arginase-1 and IL-10, a mechanism validated in preclinical studies (204, 205). Enioutina et al. (206) observed that removing MDSCs from aged splenocyte cultures restores T-cell proliferation and reduces NO levels. Moreover, Grizzle et al. (207) noted that transferring senescent MDSCs into young mice delays the immune responses against tumor cells. Mechanistically, the same study found that arginase-1 upregulation in age-associated MDSCs is a key factor limiting T-cell activity (207). Furthermore, mitochondrial DNA released by senescent tumor cells activates the cGAS-STING pathway, enhancing PMN-MDSC-driven immunosuppression and creating a feedback loop that amplifies myeloid suppression (208). In CRC and other cancers, the accumulation of senescent stromal and parenchymal cells has been linked to tumor dissemination and immune senescence (209, 210), suggesting a permissive niche for MDSC-mediated immune evasion.

### 3.2 The role of senescent adaptive immune cells in CRC

#### 3.2.1 T-cells

Aging induces thymic involution, (i) reducing naïve T-cells production while (ii) expanding memory and exhausted T-cells (211). Senescent CD8+ T-cells contribute to cancer development through reduced cytotoxicity (i.e., diminished perforin and granzyme B expression) (212) and increased exhaustion marker expression (PD-1, TIGIT, TIM-3) (213, 214). Collectively, these features diminish early tumor cell killing. Furthermore, senescent T-cells tend to adopt regulatory T-cell-like behavior, promoting anti-tumor immune suppression (215, 216). It has been reported that senescence decreases Th1/Th17 effector responses (217), thereby weakening cytotoxic support. In CRC, senescent T-cells have emerged as pivotal modulators of tumor progression, immune escape, and therapeutic resistance. Indeed, senescent phenotypes have been documented among intratumoral CD8+ T-cells and peripheral T-cell compartments in CRC patients, including increased expression of senescence markers (e.g., KLRG1, CD57, loss of CD28) alongside reduced proliferative capacity and altered cytokine secretion (218). Functionally, senescent T-cells display a SASP profile, secreting IFN-γ, IL-6, TNF-α, IL-18, and CCL5, among others (219–221). This profile may fuel chronic inflammation, recruit immunosuppressive myeloid cells, and blunt effective cytotoxic responses (222). The CCL5/CCR5 signaling pathway enhances tumor progression and cell migration, promotes angiogenesis, and drives immunosuppressive reprogramming of monocytes and myeloid cells (223). This shift leads to the development of tumorigenic macrophages and MDSCs, which in turn may also contribute to effector T-cell exhaustion (223). Additionally, IL-18, a proinflammatory cytokine of the IL-1 family that stimulates IFN-γ production, not only expands MDSCs but also strengthens their immunosuppressive functions in multiple myeloma (224). Elevated IFN-γ levels, when unaccompanied by granzyme B, further impair T-cell cytotoxic activity by upregulating immune-inhibitory molecules such as indoleamine 2,3-dioxygenase (IDO), PD-L1, and cytotoxic T-lymphocyte-associated protein 4 (CTLA-4) (225).

In elderly CRC patients, higher frequencies of senescent CD8+ T-cells and immune activation correlate with worse clinical outcomes (relapse, progression, mortality), underscoring that immune senescence restricts anti-tumor control (226). In CRC models, T-cell dysfunction is central. Tumor-derived cyclic adenosine monophosphate (cAMP) induces T-cell senescence via protein kinase A (PKA)-CREB and p38 MAPK activation, thereby promoting DNA damage and cell-cycle arrest (227). Aged CD8+ T-cells exhibit mitochondrial dysfunction, elevated ROS, and reliance on anaerobic glycolysis, impairing anti-tumor cytotoxicity in both murine CRC models and human PBMC studies (204). More mechanistically, recent experimental studies suggest that tumor-induced metabolic stress, such as lipid metabolic remodeling, accelerates T-cell senescence within the colorectal TME, thereby compromising anti-tumor functionality and reducing immunotherapy responsiveness (228).

#### 3.2.2 B-cells

Senescent B-cells may play a critical role in modulating tumor progression and the immune landscape. Indeed, these cells typically show reduced or absent proliferative potential (229), shortened telomeres (230), and expression of senescence markers such as CD27^−^IgD^−^ with elevated p16^INK4a^ or γH2AX (231–233). Aging also leads to (i) reduced peripheral blood CD19+ B-cell proportions, (ii) diminished B-cell receptor (BCR) diversity, and (iii) increased BCR clonality (234). In CRC patients, the preceding characteristics may promote a pro-tumorigenic environment through a SASP profile, which exacerbates the secretion of proinflammatory cytokines (IL-6, TNF-α) and immunoregulatory factors. Cumulative evidence indicates that senescent cells, including B-cells, may drive chronic inflammation in the gut mucosa, thereby facilitating malignant transformation and tumor progression (235, 236). Within the TME, regulatory B-cells may promote cancer progression by secreting IL-10 (237). IL-10 production may suppress inflammatory cytokine secretion (including TNF-α and IFN-γ) from cytotoxic T-cells, thereby promoting tumor growth (238). B-cells may also indirectly support tumor growth by accumulating age-associated B-cells and producing autoantibodies, which form immune complexes that activate complement and remodel tissue (239, 240). Additionally, Frasca et al. (241) reported that senescent B-cells generate fewer high-affinity antibodies, potentially impairing tumor antigen recognition and hindering tumor cell elimination. Furthermore, within the TME, senescent B-cells interact with Tregs and MDSCs, thereby amplifying immunosuppressive pathways that enable immune evasion (242).

### 3.3 Immunosenescence may modulate CRC through the gut microbiome

With age, both adaptive and innate immunity decline and become dysregulated. This immune weakening favors opportunistic bacterial expansion while depleting beneficial commensals, leading to gut dysbiosis. For example, age-related expansion of proinflammatory bacteria such as *Fusobacterium nucleatum* and *Enterotoxigenic Bacteroides fragilis* (ETBF) is frequently observed in CRC patients (243). These bacteria may drive carcinogenesis by releasing virulence factors (FadA, Fap2, RadD, *Bacteroides fragilis* toxin (BFT)) (244, 245) and activating NF-κB (246, 247) and Wnt signaling (248) in epithelial cells. Senescent immune cells adopt a SASP phenotype, releasing proinflammatory cytokines (IL-6, IL-1β, TNF-α) and chemokines. This chronic, low-grade inflammaging disrupts mucosal barriers and alters gut microbiota composition. To illustrate this, elevated IL-6 levels in aged or CRC-prone individuals promote barrier dysfunction and favor microbial translocation (249). Conversely, gut dysbiosis further exacerbates immunosenescence (250, 251). This bidirectional crosstalk likely promotes CRC. Indeed, fecal transplants from CRC patients to germ-free and conventional mice recapitulate CRC-prone phenotypes. These include an increased number of intestinal polyps and alterations in the gut microbiome, such as reduced bacterial diversity, marked depletion of beneficial species (e.g., *F. prausnitzii*), and significant enrichment of pathogenic bacteria (e.g., *B. fragilis*) (252). Additionally, the transplant leads to elevated expression of proinflammatory markers [including C-X-C motif chemokine receptor 1 (CXCR1), CXCR2, IL17A, IL22, and IL23A] and enhanced immune cell infiltration, with Th1 cells increasing from 0.44% to 2.25% and Th17 cells from 0.31% to 2.08% (252). Clinically, hypomethylation of immune-related genes (e.g., IL12RB1, MARCO) in CRC tissues correlates with aging and poor prognosis, reflecting epigenetic deregulation of immune surveillance (253, 254).

In addition to cellular changes, immunosenescence also affects humoral immunity. Evidence shows that IgA normally coats mucosal surfaces, limiting pathogenic bacterial overgrowth (255). However, age-related IgA decline allows harmful bacteria to overgrow and colonize mucosal surfaces. Notably, reduced IgA levels in older adults correlate with increased *Escherichia coli* (89, 256) and *Bacteroides fragilis* (257), both implicated in CRC initiation via colibactin-induced DNA damage (88).

Senescent Tregs and DCs are dysfunctional and may disrupt tolerance to gut antigens (258, 259). Consequently, immunosenescence may drive aberrant immune activation against commensal microbes, encouraging the selection and expansion of pro-carcinogenic species. For example, *Enterococcus faecalis* often overgrows due to age-related immune decline and is frequently elevated in CRC patients feces (260). There, it may (i) produce DNA-damaging metabolites (reactive oxygen and nitrogen species) (261) and (ii) activate oncogenes or inactivate tumor-suppressor genes (262, 263).

Lastly, senescence-related dysbiosis reduces protective short-chain fatty acids (SCFAs; e.g., butyric acid, acetic acid, and propionic acid), which normally support FOXP3+ Treg immunosuppressive function (264) and promote epithelial repair (21). Specifically, SCFAs inhibit histone deacetylases (HDACs) in neutrophils, thereby suppressing NF-κB signaling by reducing TNF and nitric oxide production (21). In macrophages, SCFAs downregulate IL-6, IL-12, and nitric oxide in the gut (265). Reduced butyrate-producing *Faecalibacterium prausnitzii* (a member of *Clostridium cluster IV)* in CRC patients may correlate with impaired mucosal immunity (266). Indeed, normally *F. prausnitzii* supports CD4+ T-cell differentiation into CD4CD8αα T-cells (Tregs) (267) in the colonic mucosa while preventing excessive inflammatory responses. Touchefeu et al. (268) showed that *F. prausnitzii* and CD4CD8αα T-cells are concomitantly depleted in CRC patients, suggesting their involvement in colonic carcinogenesis. Simultaneously, pathogenic bacteria increase production of carcinogenic metabolites (secondary bile acids, nitrosamines, and inositol-1,4,5-triphosphate). For example, *E. coli* stimulates inositol-1,4,5-triphosphate production, which promotes regeneration following intestinal damage by regulating IEC proliferation (269). Additionally, overabundance of *Clostridium cluster XI* contributes to secondary bile acid accumulation (270), which promotes DNA damage leading to CRC (271).

**Figure 4:**
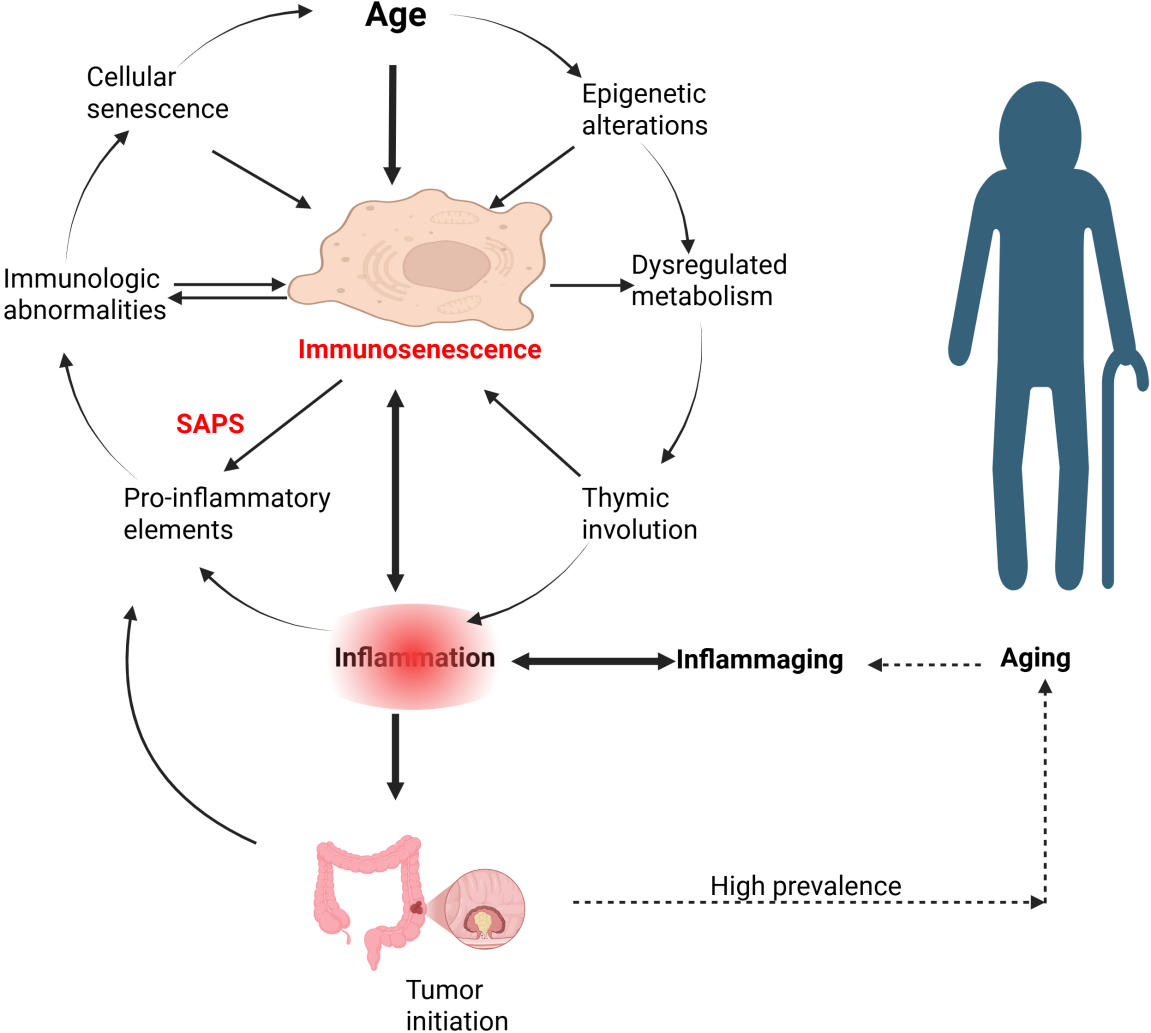
Synergistic roles of inflammaging and immunosenescence in inflammation-driven, age-associated colorectal cancer.

Inflammation and immunosenescence are intricately linked in CRC development (**Figure 4**). Extensive strategies that mitigate immunosenescence and/or inflammaging, as described in previous work such as those by Wang and Tang (18), Schmitt and Greten (21), and others (155, 272), may well be used to address or prevent CRC in aging populations. For example, IL-22, a STAT3-activating cytokine, triggers DNA damage response gene transcription to counteract inflammation-induced genotoxic effects (273). Thus, IL-22 may be a potential therapeutic agent for older individuals with chronic inflammation to reduce CRC risk. Anti-inflammatory cytokines such as IL-10 may suppress PI3K, an essential enzyme in the PI3K/AKT/mTOR pathway, thereby reducing AKT phosphorylation and activation involved in cancer development. Additionally, cetuximab combined with IL-2 or IL-15 may restore NK cell function and activation; in turn, these cells may recruit T-cells and exert anti-tumor activity (168). Senolytic drugs (e.g., fisetin) reduce SASP factors in murine CRC models, while NAD+ boosters such as nicotinamide mononucleotide (NMN) restore T-cell metabolism in elderly patients, highlighting translational potential (204, 274). Finally, gut microbiome supplementation with beneficial bacteria such as *Akkermansia muciniphila* and *F. prausnitzii* may provide substantial advantages to aging individuals. Their presence may influence the local immune response in the CRC TME (275), as they are positively associated with infiltration of anti-tumor immune cells (cytotoxic T-cells and DCs) (276, 277).

## 4 Conclusion

This review explores the combined influences of inflammaging and immunosenescence on the modulation of immune cell populations (both innate and adaptive), their underlying cellular mechanisms, and alterations to the gut-associated microbiome in relation to CRC initiation and progression. It is known that aging represents a key biological determinant in CRC pathogenesis, exerting its influence primarily through the dual processes of inflammaging and immunosenescence. Thus, on the one hand, the chronic, low-grade inflammation characteristic of inflammaging promotes the acquisition of proinflammatory phenotypes in both innate and adaptive immune cells. This, in turn, fosters a tumor-promoting microenvironment by sustaining oxidative stress, inducing DNA damage, and perpetuating aberrant cytokine signaling favoring proliferative stimuli. Initially, these proliferative stimuli are intended to support tissue repair; however, over time, they drive unregulated cellular proliferation within the colonic epithelium. On the other hand, immunosenescence is known to compromise immune surveillance and anti-tumor immunity. It drives cellular exhaustion and dysfunction while promoting the SASP, thereby contributing to the establishment of a tumor-permissive microenvironment in which immune cells are unable to effectively execute their usual and necessary regulatory and anti-tumor functions. Furthermore, the present work also reports that in aging populations, the tandem processes of inflammaging and immunosenescence profoundly influence the gut microbiome composition. Specifically, these age-associated immune alterations compromise intestinal barrier integrity, diminish beneficial microbial populations, and promote the expansion of pathogenic species. Collectively, these changes sustain and amplify chronic inflammation within the colon. Consequently, this proinflammatory milieu drives the recruitment and infiltration of exhausted, senescent, and functionally impaired immune cells into the tumor microenvironment, thereby fostering conditions that permit colorectal cancer (CRC) initiation and progression. Together, and at different levels, inflammaging and immunosenescence establish a permissive framework for malignant transformation, progression, and therapeutic resistance in the elderly.

Despite growing evidence linking these age-associated immune alterations, namely inflammaging and immunosenescence, to CRC, further work remains necessary to fully explore this vast and intriguing topic. As such, future studies integrating immunology, microbiome research, and molecular oncology are warranted to unravel the fundamental manner in which aging-driven immune dysregulation intersects with tumor biology. An understanding of this may enable the development of precision strategies (ranging from immunomodulatory interventions to early diagnostic biomarkers) aimed at mitigating the disproportionate CRC burden among aging populations.
